# Global Health Education Programs in the Americas: A Scoping Review

**DOI:** 10.5334/aogh.2745

**Published:** 2020-04-21

**Authors:** Isabel Amélia Costa Mendes, Carla Aparecida Arena Ventura, Artur Acelino Francisco Luz Nunes Queiroz, Álvaro Francisco Lopes de Sousa

**Affiliations:** 1 University of São Paulo at Ribeirão Preto College of Nursing, BR; 2 Global Health and Tropical Diseases (GHTM), PT; 3 Instituto de Higiene e Medicina Tropical, Lisboa, PT

## Abstract

**Background:**

The interest in Global Health (GH) as an academic discipline has increased in the last decade. This article reports the findings of a scoping review of studies about Global Health education in the Americas.

**Objective:**

To analyse educational programs on global health in the Americas.

**Method:**

Five electronic databases were used in a scoping review: PubMed, Scopus, Web of Knowledge, CINAHL, and Lilacs. Data collection happened in November 2017–March 2018. The following stages were rigorously observed: identification, selection, charting, and summarizing the studies that were included. To process the data, we used IRaMuTeQ software.

**Findings:**

Forty-six studies were identified and organized in categories: 1) diversity of the topics addressed in GH; 2) models of teaching; 3) emotional, cultural, and collaborative aspects in teaching GH; 4) student preparation for GH experiences; and 5) structures required for a GH course.

**Conclusions:**

The existing global health curriculum in the Americas is diffuse and limited, with a greater focus on clinical aspects. Thus, a minimum curriculum for students from different areas is needed. Results evidenced that the teaching of global health in the Americas is still incipient, although it is promising. The lack of a common curriculum for the courses in the region makes it difficult to train sensitive and capable professionals to achieve the 2030 Sustainable Development Goals.

## Introduction

Global Health (GH) refers to issues that transcend borders and national governments, demanding actions from global powers, which determine the health of different communities [1]. It involves knowledge, teaching, practice, and research on extraterritorial health issues which extrapolate national geographic borders [2]. GH teaching involves integrated courses, which can be offered within social sciences, considering the transnational aspects of health issues [3,4] while valuing the context and local capacity to act.

In the Americas, the interest in GH as an academic discipline has increased in the last decade. This is due to the growing acknowledgement of GH as a fundamental issue in health training, enabling undergraduate and graduate students to better understand the global priority agenda and its demands [5]. Human resources are central aspects for the success of GH strategies; however, there is no consensus on how to plan experiential activities to prepare students to act locally and globally and to serve as global health professionals. Furthermore, cultural diversities in the Americas (North, Central, and South) enrich GH teaching and may become a challenge for the different actors involved in these processes.

This study aims to analyse approaches, characteristics, and challenges faced by educational programs on global health in the Americas.

## Methods

This is a scoping review, with the aim of mapping relevant scientific production and gaps on a specific theme, in this case, education of health professionals [6], following this sequence: 1) identifying the research question; 2) finding relevant studies; 3) selecting the studies; 4) organizing the data; and 5) collating, summarizing, and reporting the results, according to The Joanna Brigs Institute for Scoping Reviews. The search strategy used was PICo (P: problem/population; I: intervention; Co: context), in which P: educational programs on global health; I: approaches, characteristics, and challenges; Co: Americas. This resulted in the following research question: What are the approaches, characteristics, and challenges faced by educational programs on global health in the Americas?

The inclusion criteria were: primary studies, full text available in Spanish, Portuguese, or English and published up to November 2017. Book chapters, Master’s and doctoral final reports, as well as other technical reports were included in the initial search. Data collection took place between November 2017 and March 2018.

Initially, the aim was to evaluate GH in nursing education. A generic search in electronic databases such as MEDLINE and Scielo, however, revealed few studies on this discipline (n = 3/4.5%). Therefore, the search was expanded to courses in different health disciplines. The method that was chosen permits this change in strategy [7]. After this definition, the search was based on relevant descriptors and keywords. According to the databases used, the final set of descriptors was as follows:

- PubMed, Scopus, and Web of Knowledge (MeSH descriptors): *global health AND education*.
- CINAHL (CINAHL titles): *global health OR international health AND education*.
- LILACS (descriptors and keywords): *global health AND education*, and corresponding terms in Portuguese and Spanish.

The selected studies were analysed in three phases, described below:

1. The titles and abstracts were read, followed by the application of inclusion and exclusion criteria. For articles with no abstracts, or if the abstracts did not permit the exclusion or inclusion, the articles were read. In total, 4,927 studies were extracted from PubMed, 41 from CINAHL, 609 from LILACS, and 1,001 from Web of Knowledge, totalling 6,578 studies. Two authors separately searched and analyzed the articles. In case of a lack of consensus regarding the inclusion or exclusion of an article, a third researcher was consulted.
2. There were 1,614 duplicate studies excluded, resulting in 4,964 articles. The studies were analyzed, evaluating their direct relationship to the research question in this first stage through the reading of titles and abstracts. As a result, 4,512 studies were excluded.
3. The full texts of the 452 articles were read, and 46 articles were finally selected for this study. Of those, we collected specific data, along with the method, type of investigation, outcomes, objectives, sample, results, and conclusions which were used to form the corpus to be processed, with 10,103 words derived from the original results of the papers.

Textual analysis, or lexical analysis, was used and based on transcribed verbal material. Considering the high number of selected articles and to keep the quality of the analysis process, the software IRaMuTeQ (R pour les Interface Analyses Multidimensionnelles de Textes et Questionnaires) was used to process and analyze the data. IRaMuTeQ is software extensively used to develop lexical analysis, developed under the open-source logic, based on the statistical environment of R software and python language (www.python.org) to perform different statistical analysis about the textual corpus [8,9]. The program enables different types of textual analysis, from basic flexography (calculation of the frequency of words) to multivariate analysis (hierarchical descendent classification, similitude analysis) [9,10,11].

The findings of the selected studies were grouped and formed the textual corpus, and afterwards they were analyzed using the hierarchical descending classification (HDC). This method classifies segments of text based on the vocabularies, and its grouping is based on the frequency of reduced forms (lexical radical). This analysis identifies the concurrences between the words and then shows the indications of the connectivity between the terms, which reveals the structure of a textual corpus, assigning the common elements and specificities according to the illustrative variables in the analysis [10,11,12]. In this process, Initial Context units (ICU) or Textual segments are transformed in Elementary Context Units (ECU), corresponding to the regrouping of text segments [9,12].

The software interface enables the recovery, in the original corpus, of text segments associated to each class; this is the moment in which the statistically significant word context is obtained, enabling the qualitative analysis of the data [10,11,12].

## Results

After selecting the study sample (n = 46), the publication year of the articles was verified; the majority was published more recently: 2016 (24.32%) and 2015 (18.91%). The United States stood out with 70.27% of the studies published. Studies from other countries were also published in English. There was little diversity in the study population with 45% of studies focusing on medical students. Students from the fields of nursing, public health, dentistry, nutrition, and pharmacy were also included.

The software processing generated 257 ECUs, divided into 5 classes with a final use of the corpus of 91.46%. Then, the textual fields were identified and qualitative analyzed by the researchers and the meanings were interpreted, titling them with their senses into categories: Class 1 – student’s preparation for GH experiences; Class 2 – emotional, cultural, and collaborative aspects in teaching GH; Class 3 – structures required for a GH course; Class 4 – models of teaching; and Class 5 – diversity of the topics addressed in GH.

The first division in the corpus generated two classes (1 and 2): the first one related to basic aspects for the development of courses and the other, which amplifies these aspects and expectations and challenges faculty experience when teaching this subject.

**Figure 1:**
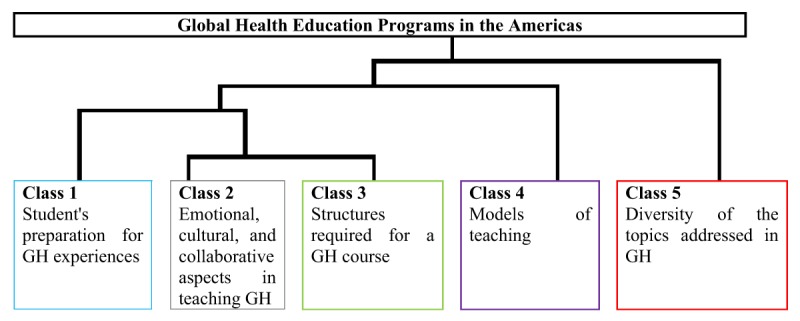
Thematic structure of the contents related to Global Health education programs in the Americas region.

The second division generated the third class, which points out the necessary structure for the course to have the desired level of quality. Class 4 appeared in the third stage suggesting that the teaching models offer a basis to deal with challenges and potentials presented before. Last, Class 5 offers multiple subjects that are present in the teaching of Global Health, and the challenges they represent to the structure (classes 1, 2, 3, and 4) already established. Thus, the class structure shows that to develop a Global Health curriculum: students and teachers need preparation and the course needs to be structured to respond to the challenges Global Health teaching raises, within a theoretical framework to support effective teaching, especially considering the great number of disciplines taught in these Global Health curricula (Figure 1).

### Class 1 – Students’ preparation for GH experiences

The preparation of students for GH experiences proved to be a major theme in the studies (19.16% of the analyzed corpus). Contents focused on the specificities of each place and course. Teachers need to do much more than mere knowledge transmission to offer a learning environment for GH that enables students to use local experiences, developing tools to cope with ethical and emotional difficulties that transcend the territorial barriers of their local reality [13,14].

For the teacher, in general, studies show that this preparation is even more challenging, as students [15,16] need to exchange information, experiences, and flexibility beyond traditional teaching standards. For this, teachers and students need to learn how to optimize existing resources and work as a team with other professions, reinforcing the importance of cross-disciplinary work [17,18,19].

The available learning resources and their capacity of innovation strongly influence the students’ skills and performance. Thus, the use of teleconferences is presented as an effective and cost-effective option to prepare students through theoretical classes, or to exchange experiences with other students, although it does not completely replace the teacher’s role [20,21].

### Class 2 – Emotional, cultural, and collaborative aspects in teaching GH

Generally, health schools in Western courses tend to be focused on clinical and procedural aspects [22,23,24], which distract and hinder students from developing skills aimed at communicating or understanding different cultures [25]. For the development of communication skills, real-world experiences seem to be the best approach to develop this competence among students. As a result, they will be able to understand the challenges and barriers that exist in advocating for public health. Innovative forms of teaching, such as case studies, group dynamics, research projects, and real-world experiences are also effective in positioning students to be able to exercise their leadership skills [15].

These findings explain and validate the interdependence between classes 1 and 2, according to the adopted method, as in the first the problem is presented and in the second, some solutions are adopted by schools/programs.

As a complementary aspect of student preparation, our findings point to teaching emotional, cultural, and collaborative skills to teach and develop the values and principles of team-building to plan and execute the project goals. This experience allows the student to apply leadership tools that support collaborative practice, through values and principles of respect for cultural diversity [17].

Stimulating inter-professional values and communication skills that demonstrate respect, empathy, and awareness of unique cultures is a way to value roles, responsibilities, and knowledge represented by other professionals and groups that work in global health [17,22,23].

### Class 3 – Structures required for a GH course

This class, the largest of all (with 25.3% of the corpus), comprehends the themes that focus on the minimum requirements for the establishment of a GH course in the institution. One of the most important points was the students’ debriefing [15,24,25]. This aspect, even if it demands time and organization from the institution that intends to send its students abroad, has significant benefits in understanding students’ ethical and cultural aspects.

Curriculum development appears as one of the crucial aspects of GH activities. To structure the course, the curriculum should not only meet the educational needs of the students but also the clinical and social demands of the partner countries, as global health refers to the scope of the problems, not its localization [26]. Interdisciplinarity is also an important aspect to be incorporated, as western medical students receive relatively few tools to deal successfully with potential ethical dilemmas [27,28,29]. For this development, studies [19,30] suggest that the GH curriculum may be similar to the accreditation process for undergraduate medical and other public health disciplines.

One tool considered to be extremely positive, as evaluated by the students, was the use of distance-learning technologies. These technologies allowed the simultaneous teaching of classes in different countries and the interaction between students of different backgrounds during theoretical activities [18,31].

The partnership among universities (two or more), such as in undergraduate programs or multiple degrees, was pointed out as a success factor in cases reported in this review [13,32,33]. This partnership should not only take into account bureaucratic and systematic aspects but also promote concordance between the cultural differences involved in the experience, valuing local aspects.

### Class 4 – Models of teaching

Following the prior classes, which approached more technical aspects related to GH teaching, this class focuses on theoretical-methodological aspects. Given the multiplicity of themes and factors to be considered in GH education, many studies provide models of courses and curricula to disseminate their experiences, facilitate the construction of new courses [34,35] and/or contribute to an overview of existing courses.

As an example, one study [36] used the Delphi technique to build a series of skills necessary for its students, among them: capacity strengthening; collaborating and partnering; ethical reasoning; health equity; and socio-cultural and political awareness, among others.

Studies point out that students rarely had previous international experience and, while visiting other communities, they were able to experience much greater freedom in their GH courses than in their countries of origin [27,37].

The models show that the best performing courses presented a hybrid structure of theoretical classes and international practical experiences [19,30]. Those courses that have a clear and structured curriculum, faculty members, and international partnerships report having more financial resources to send their students abroad, as well as to structure preparation and debriefing sessions [18,30,31]. Student exchange is rarely two-way though [31,36,37]. This one-way exchange impairs more structured global health experiences, able to connect specific strengths of different institutions.

As a more feasible and flexible option for the implementation of new GH courses studies that analyzed GH education for pharmacology [19,38] suggest that elective coursework in didactic education can be used to prepare students to engage in international or global experiences. Thus, students more interested in the subject can seek this information in a way that is reconciled with graduation timelines.

### Class 5 – Diversity of the topics addressed in GH

Class 5 is shown as the central theme of our findings, as it brings a common aspect to all the studies: the complexity of topics involved in GH teaching. This demonstrates how GH is a versatile and helpful subject in student education. The understanding of problems at the international level provides a deeper appreciation of global public health issues and increases cultural awareness and understanding in clinical practice [13,39].

The teaching of GH was indicated as the key strategy to building a high degree of synergy among global partners, as natural linkages exist through a common understanding of the profession [33] for the development of future leaders [40,41].

Strategies of global health education in the U.S. can be classified into three different models: integrated global health tracks; global health electives; and additional research years [18,23]. The implementation of global health training within these programs requires continued optimization, but this training is essential to prepare the next generation of health professionals to address the global aspects of specific diseases, along with purely clinical aspects [41].

One important skill that can be transferred during GH courses is the ability to interpret and visualize metrics used to characterize global health problems, including mortality and incidence health rates in a specified community, country, or region [16].

## Discussion

Educational programs on global health in the Americas are incipient, yet promising. The complexity of the theme, as well as the social and cultural differences between countries considerably affect teaching and make it difficult to establish a panorama of the initiatives that are present on the continent. Thus, this manuscript is pioneering in portraying this reality in the Americas through a review.

Also, the basic structure at the curriculum level for students from different health areas is necessary considering that global health also corresponds to collaborative transnational research and action to promote health for all, and it not restricted to one area or specific courses [42,43,44].

The results showed that this curriculum needs to go beyond the technical knowledge regarding each discipline, expanding its coverage to potential ethical issues that arise from relationships at several levels [45]. At the macro level, it is important to better understand the underlying power relations between institutions and faculty members from developed and underdeveloped countries in the negotiation of these programs. At the meso-level, it is crucial to consider the relationships emerging from the interaction between students from developed and underdeveloped countries during the implementation of global health courses. At the micro-level, these power imbalances reflect on the relationship of these students with the community that they should serve [46,47].

The appropriate inclusion of this theme, considering the different mentioned levels, has shown to bring benefits for students. The literature shows that GH education can act as an important tool to facilitate students’ understanding of the global burden of diseases, health challenges and disparities, current trends and economic risk factors [32]. This is an important aspect of how GH courses can be used to improve the training of different professions as a whole and not only as an isolated aspect of their curriculum. Global health training can offer important benefits for health students and their broader understanding of health inequities, as well as of their roles in dealing with these issues, playing a strategic role to strengthen health systems on the path towards universal health coverage [48].

Debriefing appeared as a powerful and core aspect to cope with ethical challenges, enabling a continuing learning process for all the students and faculty. It facilitates the exchange of different perspectives regarding the same issue [49,50]. This tool is limited to academic scenarios though, as it does not involve the community. For an improvement in the preparation of their students, GH courses should also consider the use of other tools to enable the participation of communities and underrepresented populations in all phases of these courses, from preparation to evaluation [51]. These policies need to be internalized by the institutions involved in these programs, provoking cultural transformations, which value open communication strategies, stimulating active participation from the different partners [26].

A fundamental aspect of global health training is the focus on preparing the next generation of health professionals. Several studies found in this review addressed the need for broad preparation involving the logistics of the programs, but also providing tools that can enable students to better deal with different world-views, delicate subjects, and cultural differences [48,49,50,51,52].

The immersion of students in communities from low- and middle-income countries, for example, may allow them to confront their expectations about the clinical caregivers, considering the reality of underprivileged and vulnerable communities [13,42].

These experiences can overcome the fragility of the western education of health students, which is extremely focused on clinical aspects and can result in ethical and anthropological conflicts. Coping with the conflicts can improve students’ understanding of the situations, though, and enable collaborative projects that may lead to professional and personal growth [22,32,33].

As international clinical education is fraught with ethical, pedagogical, and logistical issues that are difficult to understand and require ongoing analysis and management, some institutions make their curricula available as open and free to be a model and facilitate the implementation of the course in other institutions or countries [17,45].

There was no mention of the preparation of local students to receive foreign students in their environment, though. This should also be a concern for both parties involved in these courses, as it is a source of ethical problems. If these issues are openly discussed and analyzed at the different participant levels, providing a joint decision-making process, this may enable a culture of transformation that will include not only the ones directly participating in these courses, but will also be shared by a broader range of members of these communities [53].

The development of cultural and emotional competencies is implicit as the basis for the success of Global Health courses. Therefore, the investment in the development of these skills must value interpersonal relationships and the use of effective verbal and non-verbal communication tools, considering cultural differences [53,54]. Global health communication is complex, involving cultural, social, legal, and political diversity [53], which needs to be taken into account in the preparation of students, faculties, and communities.

This complexity is reflected in the difficulty reported in several studies to create a model for teaching global health. Studies demonstrate that this may be unachievable, especially considering that institutions are culturally different. Some publications list core aspects that have to be considered in the design and development of global health courses though [19,30].

Our research has limitations. The concentration of cross-sectional research reveals that little is known about the longitudinal effects of the GH courses. In addition, comparing such different countries (in terms of culture, income, and education) makes it difficult to establish a panorama, as well as to propose public policies.

## Conclusion

The existing global health curriculum in the Americas is diffuse and limited, what seems to reflect the region’s social and economic differences, with a predominance of initiatives from North America, especially the United States.

The findings show that this is related to the complexity, depth, and intensity that the theme requires from students, teachers, and institutions. Therefore, a minimum structure involves structure components (such as transdisciplinarity, teaching innovative tools, the establishment of meaningful partnerships) and theoretical-methodological aspects (including a clear and structured curriculum, a consistent alignment with countries’ priorities, needs, and preferences as well as the reciprocity as hosts). Therefore, there is a need for more comprehensive content, considering the characteristics and differences of the countries in the region.

Thus, a minimum curriculum is required for students from different fields. In addition to the technical knowledge of each subject, this curriculum needs to include possible ethical issues that arise from relationships at various levels. In sum, considering the following ten years for the accomplishment of the Sustainable Development Goals (2030 Agenda), the investment in human resources for health is an essential component for the improvement of the health systems globally.
